# Molecular Mechanism of Exogenous ABA to Enhance UV-B Resistance in *Rhododendron chrysanthum* Pall. by Modulating Flavonoid Accumulation

**DOI:** 10.3390/ijms25105248

**Published:** 2024-05-11

**Authors:** Wang Yu, Fushuai Gong, Hongwei Xu, Xiaofu Zhou

**Affiliations:** Jilin Provincial Key Laboratory of Plant Resource Science and Green Production, Jilin Normal University, Siping 136000, China

**Keywords:** metabolomics, transcriptomics, *R. chrysanthum*, ABA, flavonoid

## Abstract

With the depletion of the ozone layer, the intensity of ultraviolet B (UV-B) radiation reaching the Earth’s surface increases, which in turn causes significant stress to plants and affects all aspects of plant growth and development. The aim of this study was to investigate the mechanism of response to UV-B radiation in the endemic species of *Rhododendron chrysanthum* Pall. (*R. chrysanthum*) in the Changbai Mountains and to study how exogenous ABA regulates the response of *R. chrysanthum* to UV-B stress. The results of chlorophyll fluorescence images and OJIP kinetic curves showed that UV-B radiation damaged the PSII photosystem of *R. chrysanthum*, and exogenous ABA could alleviate this damage to some extent. A total of 2148 metabolites were detected by metabolomics, of which flavonoids accounted for the highest number (487, or 22.67%). KEGG enrichment analysis of flavonoids that showed differential accumulation by UV-B radiation and exogenous ABA revealed that flavonoid biosynthesis and flavone and flavonol biosynthesis were significantly altered. GO analysis showed that most of the DEGs produced after UV-B radiation and exogenous ABA were distributed in the cellular process, cellular anatomical entity, and catalytic activity. Network analysis of key DFs and DEGs associated with flavonoid synthesis identified key flavonoids (isorhamnetin-3-O-gallate and dihydromyricetin) and genes (TRINITY_DN2213_c0_g1_i4-A1) that promote the resistance of *R. chrysanthum* to UV-B stress. In addition, multiple transcription factor families were found to be involved in the regulation of the flavonoid synthesis pathway under UV-B stress. Overall, *R. chrysanthum* actively responded to UV-B stress by regulating changes in flavonoids, especially flavones and flavonols, while exogenous ABA further enhanced its resistance to UV-B stress. The experimental results not only provide a new perspective for understanding the molecular mechanism of the response to UV-B stress in the *R. chrysanthum*, but also provide a valuable theoretical basis for future research and application in improving plant adversity tolerance.

## 1. Introduction

In recent years, there has been a gradual depletion of the ozone layer as a result of increased environmental challenges, which has led to a significant increase in ultraviolet radiation b (UV-B) in the 280–320 nm band, which in turn has increased the amount of UV radiation reaching the Earth’s surface [1,2]. In fact, UV-B stress can have profound effects on plants at multiple levels, including their morphological structure, physiological and biochemical processes, and molecular composition, and may even lead to the end of the plant’s life process [3]. It has been demonstrated that UV-B stress causes *Kobresia humilis* (C. A. Mey ex Trauvt.) Sergievskaya leaves to thicken while also causing their epidermal cells to shrink and lose water [4]. Enhanced UV-B radiation can cause crop plant growth to be stunted and dwarfed, with one study showing that winter wheat plants exposed to UV-B radiation were dwarfed by 31.3 per cent compared to a control group not exposed to UV-B radiation [5]. UV-B stress can damage the chloroplast structure of plant leaves or lead to the photooxidation of chlorophyll. These negative effects ultimately impact the photosynthetic process [6]. The effects of UV-B radiation on the plant light reaction system are also mainly reflected in the disruption of the photo-PSII reaction centers, causing a decrease in photosynthetic electron transfer efficiency and leading to the inhibition of photosynthesis [7]. Different species have varying capacities and physiological reactions to defend against and repair damage induced by UV-B radiation in an environment where UV-B radiation is still increasing globally. Consequently, plants have progressively evolved their distinct defense mechanisms to counteract the damaging effects of UV-B radiation [8].

*Rhododendron chrysanthum* Pall. (*R. chrysanthum*), popularly called cowhide tea, is an uncommon plant that is highly valuable for its ornamental and medicinal uses. This plant, which is native to China’s Changbai Mountains, is also a vital and invaluable resource for researchers studying plant stress tolerance [9]. Plants invariably come into contact with a range of environmental pressures since they are sedentary and cannot move as easily as animals to avoid dangers [10]. Since *R. chrysanthum* grow year-round in harsh environments with high altitude, low temperatures, and strong UV-B radiation, they have gradually evolved a special set of mechanisms for resisting stress during their long evolutionary process. Acetylation proteomic studies have revealed UV-B’s ability to hinder *R. chrysanthum*’s photosynthesis, with the acetylation of PSII protein noted in this process. Nonetheless, the acetylated PSII protein may aid *R. chrysanthum* in enhancing its tolerance to UV-B radiation to a certain degree [11]. Joint proteomic and transcriptomic studies have shown that *R. chrysanthum* is able to withstand the effects of low temperatures by eliciting a number of responses through a regulatory network consisting of Ca^2+^ signaling, ABA, and MAPK cascades [12]. However, studies on the response of secondary metabolites to abiotic stresses in chrysanthemum are still scarce, and it is significant to deeply explore the resistance mechanism of chrysanthemum from the perspective of secondary metabolism, which is worthy of further exploration.

Flavonoids are the most abundant class of phenolic water-soluble pigment substances in plants. Flavonoids, a key class of secondary metabolites in plants, are synthesized from the phenylalanine pathway. Its basic structure consists of two benzene rings joined through a linkage containing three carbon atoms (i.e., C6-C3-C6), forming a series of compounds with this basic backbone [13,14]. Flavonoids play an indispensable role in plant growth and development processes and in response to abiotic stresses such as UV-B radiation, low temperature, and drought [15,16]. When plants are stimulated by these abiotic stresses, they form, transform, and accumulate flavonoid compounds with strong antioxidant activity. These flavonoids are able to scavenge reactive oxygen species (ROS) produced as a result of stress, thereby enhancing plant survival [17]. In response to adversity strain, the flavonoid content in plants responds positively to UV-B radiation stress, but the magnitude of the change in response varies by species, cultivar, and irradiation measure [18]. Numerous studies have shown that different plants have their own unique flavonoid regulatory mechanisms in response to UV-B stress. Therefore, more in-depth studies on the dynamic changes of flavonoids in *R. chrysanthum* under UV-B stress are still needed to further reveal its adaptive mechanisms.

Phytohormones are key signaling molecules responsible for regulating plant growth and development. When plants are faced with environmental stress, phytohormones can act quickly to help them adapt to changes in the environment and ensure their survival and growth under unfavorable conditions [19]. Within the range of hormones found in plants, ABA (abscisic acid) stands out as a crucial hormonal mediator, significantly influencing responses to stress from adversity [20]. Salt stress triggers a significant accumulation of ABA in plants. This accumulation, in turn, slows down the expansion of leaves and prompts stomatal closure. Through these mechanisms, the transpiration rate of the plant is reduced, thus effectively mitigating the damage caused by salt stress [21]. ABA has the function of regulating the antioxidant defense system in plants, and through this mechanism, ABA can help plants resist adversity stresses, such as low temperature and drought, and enhance their adversity adaptability [22]. Furthermore, ABA succeeded in triggering H_2_O_2_ buildup, subsequently boosting cucumber leaves’ antioxidant properties under Ca (NO3)_2_ stress, thus increasing their resilience to stress from adversity [23]. Previous studies have shown that ABA can enhance plant resistance to UV-B radiation through the regulation of phenolic acid [24,25]. In addition, previous studies found that exogenous ABA-treated plants were able to enhance the area and width of stomata that had declined as a result of being under UV-B stress, suggesting that ABA may enhance plant acclimation to UV-B stress by modulating stomatal properties [26]. Recognizing ABA’s vital function in responding to plant adversity, numerous experiments using external ABA were undertaken and consistently demonstrated that ABA markedly improves the plant’s ability to endure stress [27,28]. Consequently, this study delved deeper into how external ABA influences *R. chrysanthum*’s reaction to UV-B radiation, examining it from metabolomics and transcriptomics viewpoints.

Widely-targeted metabolomics analysis based on UPLC-MS/MS (ultra-performance liquid chromatography tandem mass spectrometry) has gradually become the mainstream of current research on plant responses to adversity stress from the perspective of metabolomics due to its high specificity, high detection sensitivity, and accurate quantification [29,30,31]. Transcriptomics enables the systematic study of gene function and reveals the molecular mechanisms of complex biological pathways and trait regulatory networks at the overall transcriptional level [32]. Combining metabolomics with transcriptomics offers a deeper insight into biological system alterations, allows for a collective examination of possible regulatory networks in organisms, and more thoroughly uncovers how organisms react to abiotic stress factors [33].

Despite the abundance of research on ABA, investigations into *R. chrysanthum*‘s exogenous ABA remain scarce. Therefore, in the present study, we analyzed the flavonoid dynamics of *R. chrysanthum* under UV-B and exogenous ABA treatments by widely-targeted metabolomics analysis based on UPLC-MS/MS and transcriptomics jointly. This provides a theoretical reference for understanding how flavonoids respond to abiotic stresses and the role of external ABA in enhancing plant stress tolerance.

## 2. Results

### 2.1. Damage Caused by UV-B Radiation to the R. chrysanthum Photosystem and the Mitigating Effect of Exogenous ABA

**Figure 1:**
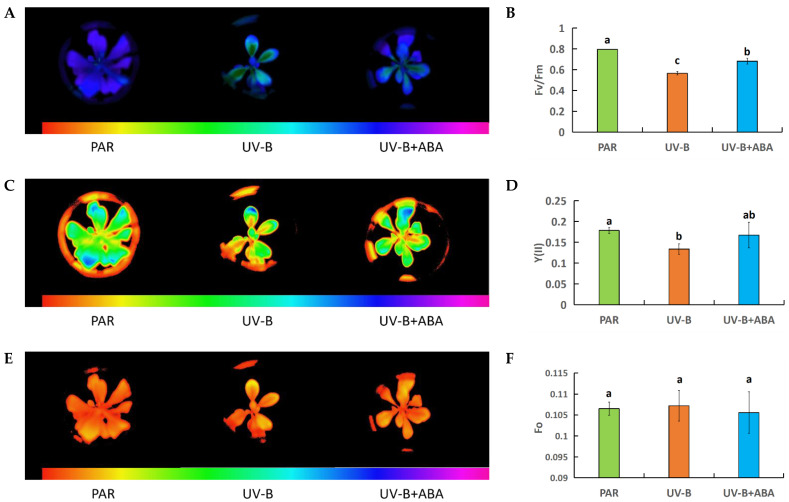
Chlorophyll fluorescence images of *R. chrysanthum* photosystem II (PS II) under different treatments. The images (**A**,**C**,**E**) represent Fv/Fm, Y (II), and Fo, respectively. (**B**,**D**,**F**) are bar graphs representing average values for Fv/Fm, Y (II), and Fo, respectively. The height of each bar graph represents an average of three copies, and the length of each error bar represents a corresponding standard deviation. After the ANOVA test, the difference between different letters is significant (*p* < 0.05).

To examine the distinct impacts of UV-B radiation and external ABA on *R. chrysanthum*’s chlorophyll fluorescence imagery, both UV-B radiation and external ABA treatments were administered to *R. chrysanthum*. The results showed that UV-B radiation affected all chlorophyll fluorescence parameters (maximum efficiency of PSII photochemistry, Fv/Fm; actual efficiency of PSII photochemistry, Y (II); minimal fluorescence, Fo) to different degrees. Nonetheless, the alterations caused by UV-B radiation were somewhat lessened following the application of external ABA. The Fv/Fm and Y (II) values were decreased significantly by UV-B radiation treatment, and Fv/Fm decreased by 80% and Y (II) by 28% from pretreatment. However, after treatment with exogenous ABA, this decreasing trend was alleviated, and Fv/Fm and Y (II) recovered (Figure 1A–D; Table 1). Furthermore, the Fo value is essential for evaluating modifications to the PS II photosystem of the plant. The Fo value will exhibit an increasing trend and turn into a significant reference index, indicating damage to the PS II photosystem once the reaction center is compromised. UV-B radiation increased the Fo value. However, the UV-B-induced increase in the Fo value was mitigated to some degree after treatment with exogenous ABA, though not significantly (Figure 1E,F; Table 1).

**Figure 2:**
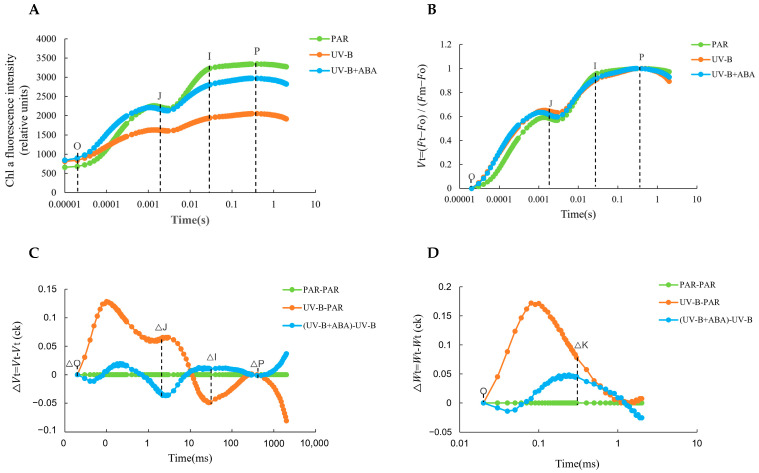
Effect of UV-B radiation and exogenous ABA on OJIP curves of *R. chrysanthum* leaves. (**A**) OJIP raw curves; (**B**) O-P point-normalised Vt curves; (**C**) The difference ΔVt between UV-B and control PAR and ABA versus UV-B curves; (**D**) The difference ΔWt between UV-B and control PAR and ABA versus UV-B curves.

The effects of UV-B radiation and exogenous ABA on the photosystem of *R. chrysanthum* were further illustrated by OJIP curves. After UV-B radiation, the fluorescence intensity of the JI and IP phases decreased successively, which indicates that UV-B radiation reduced the ability of the fast-reducing PQ library and the slow-reducing PQ library of *R. chrysanthum* to be reduced, and the receptor side of the PSII reaction center was injured. In contrast, the increase in fluorescence intensity of the JI and IP phases after the application of exogenous ABA indicates that the supply of ABA promotes the reduction of the PQ library to a certain extent and improves the electron transfer capacity (Figure 2A).

In the rapid fluorescence detection process, the differences in OJIP curves of the leaves of the *R. chrysanthum* under different treatments often also include the effects of factors other than stress, such as leaf surface appendages, pigmentation, and leaf thickness. Therefore, it is necessary to standardize the OJIP curves by mathematical methods, so that the start and end points of all curves are in a uniform state, eliminating unnecessary factors (Figure 2B–D).

The OJIP curves were normalized to obtain relative variable fluorescence (Vt)-induced kinetic curves, and at the O-J stage, the fluorescence intensity increased under UV-B stress, suggesting that UV-B radiation inhibits electron transfer on the PSII receptor side of *R. chrysanthum*. Conversely, the addition of ABA alleviated this inhibition, but the change was not significant (Figure 2B). After UV-B radiation, ΔJ > 0 indicates that QA-accumulation occurs on the receptor side of the PSII reaction center, and light energy is mostly used to reduce QA (the primary quinone acceptor), resulting in a blockage of the electron transfer process from QA to QB (the secondary quinone acceptor). A decrease in ΔJ after exogenous ABA would indicate a decrease in capacity dissipation in electron transfer, allowing more electrons to enter the transfer chain (Figure 2C). The appearance of the K phase is due to damage to the donor side of PSII and partial inhibition of the acceptor side prior to QA, resulting in a decrease in acceptor-side electron transport capacity, and this phase can be used as an indicator of damage to the OEC (oxygen evolving complex). ΔK > 0 after UV-B radiation indicates that the electron donor side of PSII is injured, with a reduced number of OECs, an increased degree of destruction, and a weaker ability to provide electrons downstream. In contrast, the magnitude of the ΔK rise decreased after exogenous ABA treatment, indicating that exogenous ABA alleviated the damage caused by UV-B on the electron donor side (Figure 2D).

In conclusion, the combined results of chlorophyll fluorescence images and OJIP curves showed that UV-B radiation was able to cause a certain degree of damage to various aspects of the PSII reaction center of *R. chrysanthum*, while the application of exogenous ABA alleviated to a certain extent the damage caused by UV-B radiation to the photosynthetic apparatus.

### 2.2. The Positive Reaction of Flavonoids against UV-B Stress of R. chrysanthum

**Figure 3:**
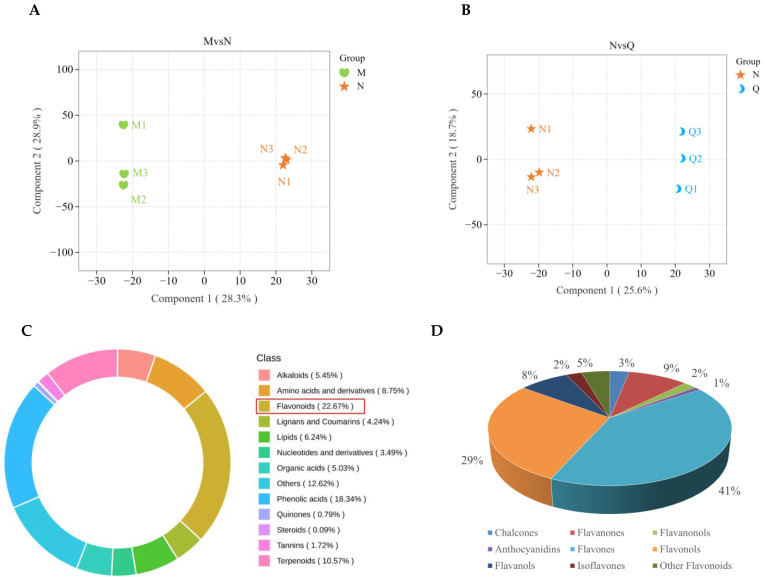
Information related to the detected metabolites of *R. chrysanthum* under different treatments. (**A**) OPLS-DA (orthogonal partial least squares discriminant analysis) of MN group metabolites; (**B**) OPLS-DA of NQ group metabolites; (**C**) 2148 metabolite classes make up the ring. Each color represents a metabolite class, and the area of the color block indicates the percentage of that class; (**D**) Statistical pie chart of the detected flavonoids and their percentages.

Further metabolite analyses were carried out using orthogonal partial least squares discriminant analysis (OPLS-DA) on three independent replicates of *R. chrysanthum* samples subjected to PAR treatment (M), UV-B radiation treatment (N), and exogenous ABA treatment (Q) (Figure 3A,B). OPLS-DA plots showed that component 1 and component 2 were 28.3% and 28.9% (25.6% and 18.7%), indicating that the three independent replicates were significantly differentiated and the individual samples were biologically reproducible after UV-B radiation treatment and exogenous ABA treatment. In addition, model validation of the OPLS-DA plots after the two treatments showed that the Q^2^ value was 0.7 after UV-B radiation and 0.643 after exogenous ABA treatment, both of which were greater than 0.5, suggesting that OPLS-DA is a valid model (Supplementary Figure S1). This indicates that the quality of the metabolomic data is reliable and can be used to further analyze the dynamics of flavonoids.

In this experiment, the widely-targeted metabolomics technique based on UPLC-MS/MS analysis was used to comprehensively examine the metabolites in *R. chrysanthum*. The results showed that a total of 2148 metabolites were detected (Supplementary Table S1). The 2148 metabolites that were found were categorized into 13 groups. Of these, the flavonoids category contained 487 compounds, which made up 22.67% of all the metabolites found, making it the most prevalent group of metabolites (Figure 3C). The 487 detected flavonoids were classified and counted into nine different categories. Among them, the most abundant category was flavones (41%), followed by flavonols (29%) and flavanones (9%) (Figure 3D). The relative amounts of these flavonoid compounds are shown in the clustered heat map (Supplementary Figure S2). All in all, there was a positive reaction from flavonoids to UV-B radiation.

### 2.3. UV-B Radiation and Exogenous ABA Affect the Flavones and Flavonols Content of R. chrysanthum

**Figure 4:**
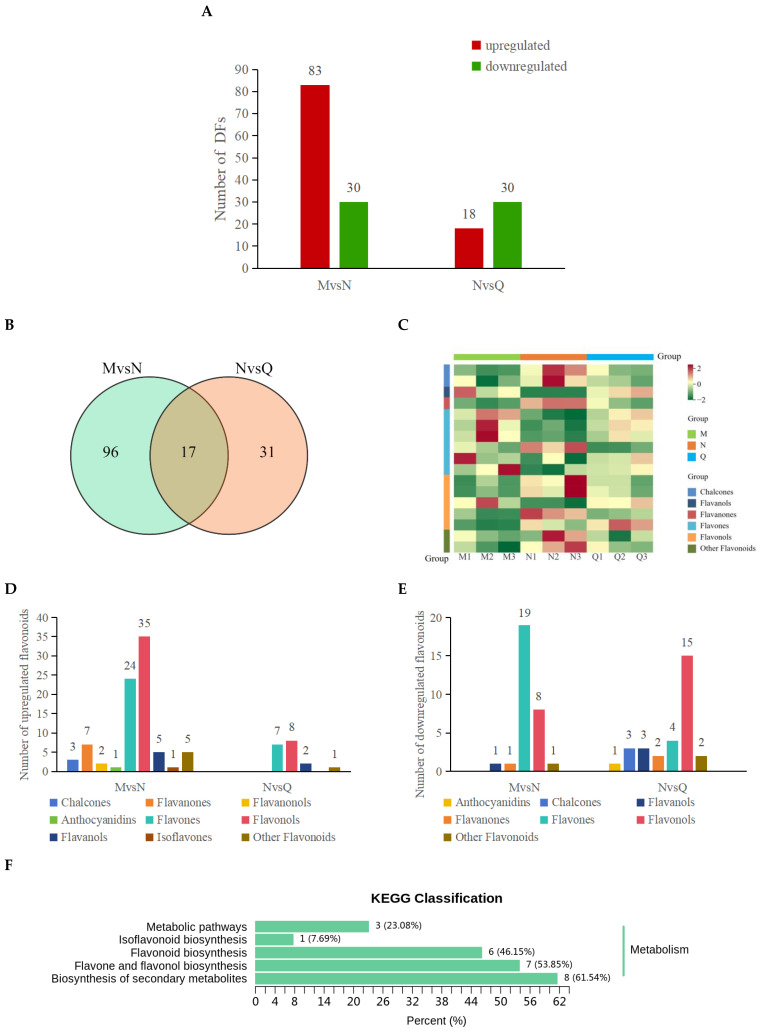

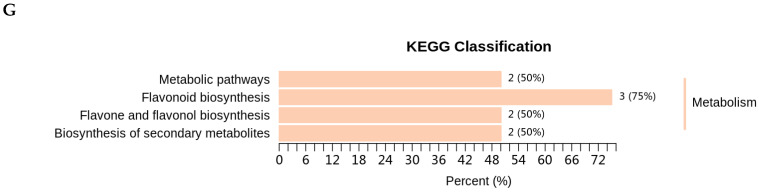
Information of DFs of *R. chrysanthum* under different treatments. (**A**) Statistics of the number of up-regulated and down-regulated DFs under UV-B and exogenous ABA treatments; (**B**) Wayne diagram of the DFs under UV-B radiation and exogenous ABA treatments; (**C**) Heatmap of clustering of the 17 common DFs; (**D**) Statistics of the number of types of up-regulated DFs; (**E**) Statistics of the number of types of down-regulated DFs; (**F**) KEGG enrichment analysis of the DFs in MvsN KEGG enrichment analysis; (**G**) KEGG enrichment analysis of DFs in NvsQ.

Differential isoflavone DFs (different flavonoids) were screened on the basis of VIP (variable importance in projection) and FC (fold change) values, and flavonoid metabolites were considered to be significantly different if they met both VIP > 1 and FC ≥ 1.5/FC ≤ 0.67. Following UV-B radiation (MvsN), *R. chrysanthum* produced 113 DFs in total, of which 30 DFs were reduced and 83 DFs were increased. After exogenous ABA treatment (NvsQ), *R. chrysanthum* produced a total of 48 DFs, of which 18 DFs were increased and 30 DFs were decreased (Figure 4A).

The Wayne diagram directly illustrates the count of both shared and distinct DFs between the two comparison groups, MvsN and NvsQ (Figure 4B). Within the 17 prevalent DFs, there were two chalcones, one flavone, three flavonols, and two additional flavonoids, with their levels notably rising post-UV-B radiation exposure but ceasing to rise following external ABA exposure. Conversely, external ABA succeeded in increasing the levels of many flavones (five species), which diminished following UV-B radiation exposure, and a minor portion of flavonols (one species) saw a reduction after UV-B radiation exposure (Figure 4C; Supplementary Table S2).

Further comparisons were made to analyze the up- and down-regulated DFs in the two comparison groups and to count the number of species. The findings indicated that exposure to UV-B radiation and the application of external ABA both led to notable variations in the levels of flavones and flavonols. UV-B radiation increased the levels of 24 flavones and 35 flavonols and decreased the levels of 19 flavones and 8 flavonols; exogenous ABA caused an increase in the levels of 7 flavones and 8 flavonols and a decrease in the levels of 4 flavones and 15 flavonols (Figure 4D,E).

KEGG enrichment analyses were performed on DFs produced in UV-B radiation treatment (MvsN) as well as exogenous ABA treatment (NvsQ). The results showed that flavonoid biosynthesis (ko00941) and flavone and flavonol biosynthesis (ko00944) were significantly altered in *R. chrysanthum* after UV-B radiation as well as exogenous ABA treatment (Figure 4F,G).

### 2.4. Transcriptomic Analysis of R. chrysanthum under UV-B and Exogenous ABA

Transcriptomics analyses were performed on *R. chrysanthum* leaf samples, and nine cDNA libraries were constructed, yielding a total of 76.65 Gb of RNA-seq data, which were assembled and de-redundant to yield 47,598 Unigenes. A total of 43.69–45.44 million raw reads were obtained. After completing cleaning and quality checking of the data, approximately 42.34–43.46 million clean reads were obtained, with Q20 ranging from 97.83% to 98.43%, Q30 ranging from 92.4% to 94.51%, and GC content ranging from 43.63% to 44.01%, which are all proof of the high degree of the sequencing results’ reliability (Table 2).

**Figure 5:**
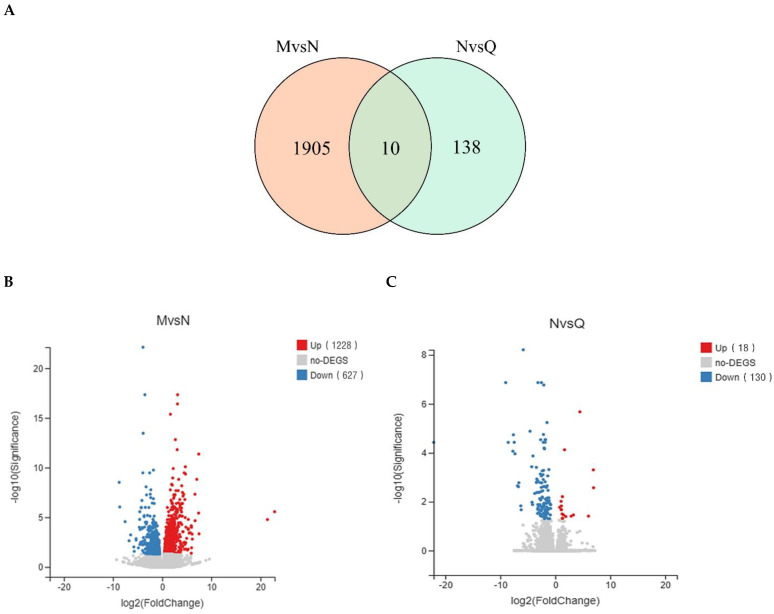
Multi-perspective analysis of differentially expressed genes (DEGs) among different controls. (**A**) Wayne plots of DEGs between Groups M and N and between Groups N and Q; (**B**) Statistical volcano plots of the number of upward and downward adjustments of DEGs in Groups M and N; (**C**) Statistical volcano plots of the number of upward and downward adjustments of DEGs in Groups N and Q.

**Figure 6:**
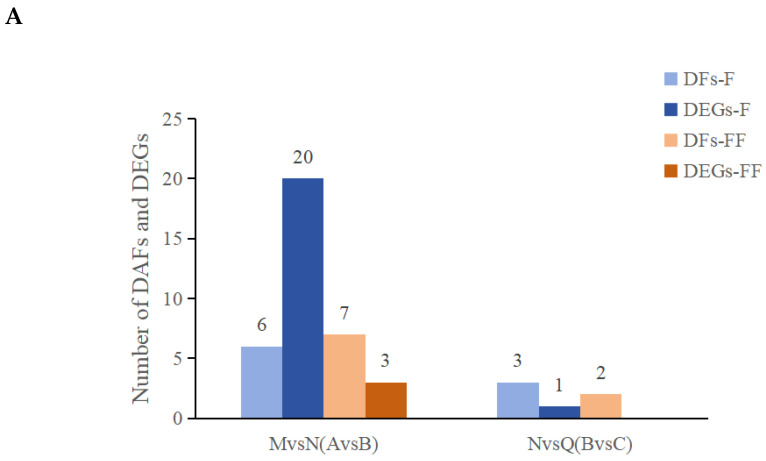

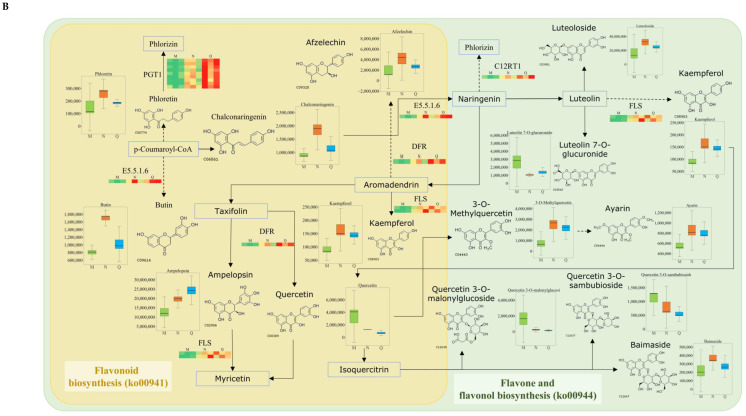
Dynamic changes of flavonoid-related pathways under two treatments. (**A**) Statistics of the number of DFs and DEGs in the two flavonoid-related pathways; (**B**) Diagram of the network pathways of DFs and DEGs in the flavonoid-related pathway.

DEGs (differentially expressed genes) were screened by Q value (adjusted *p* value) < 0.05 and FC > 1. The analysis using Venn diagrams was conducted to pinpoint genes with DEGs shared by each control group, revealing 10 DEGs shared between the two groups (Figure 5A). After UV-B radiation (MvsN) treatment, 1915 DEGs were generated, with 1288 up-regulated and 627 down-regulated (Figure 6B). After exogenous ABA treatment (NvsQ), a total of 148 DEGs were produced, of which 18 were up-regulated and 130 were down-regulated (Figure 5B,C). Distribution and expression of DEGs in the two comparison groups are shown in the clustered heatmap (Supplementary Figure S3). According to gene ontology analysis (GO), DEGs appeared predominantly in the biological process and molecular function regardless of UV-B radiation or exogenous ABA treatment (Supplementary Figure S4). The results of the analyses showed that exogenous ABA was able to alleviate the effects of UV-B on *R. chrysanthum* accordingly, thus improving the ability of *R. chrysanthum* to withstand UV-B stress.

### 2.5. Key Enzymes in the Flavonoid Synthesis Pathway Regulate the DFs Network

All DAFs and DEGs involved in the two flavonoid-related pathways were quantified (Figure 6A). The results showed that after UV-B radiation (MvsN), a total of 20 DEGs and 6 DAFs were simultaneously mapped to the flavonoid biosynthesis pathway (F, ko00941), and 3 DEGs and 7 DAFs were mapped to the flavone and flavonol biosynthesis pathways (FF, ko00944). After exogenous ABA treatment (NvsQ), 1 DEG and 3 DAFs were mapped to the flavonoid biosynthesis pathway, and 2 DAFs were mapped to the flavone and flavonol biosynthesis pathways.

To comprehensively explore the upstream and downstream relationships between DFs and DEGs, the metabolic pathway network of DFs and DEGs was constructed by mapping these key DAFs and DEGs onto KEGG pathway maps (Figure 6B). UV-B and exogenous ABA regulated the expression of DEGs encoding five enzymes involved in two flavonoid pathways (ko00941, ko00944). The DEGs encoding chalcone isomerase (E5.5.1.6, [EC: 5.5.1.6]), encoding flavanone 7-O-glucoside 2″-O-beta-L-rhamnosyltransferase (C12RT1, [EC:2.4.1.236]), and encoding dihydroflavonol 4-reductase (DFR, [EC:1.1.1.219 1.1.1.234]) all involve only one DEG, and all three DEGs change in a similar trend, being up-regulated after UV-B radiation (MvsN) and persistently up-regulated after exogenous ABA (NvsQ). The DEGs involved in encoding phlorizin synthase (PGT1, [EC: 2.4.1.357]) were more numerous (nine in total) and these DEGs also followed a similar trend to those of the other related enzymes, being up-regulated after UV-B radiation and persistently up-regulated after exogenous ABA. There are two types of DEGs encoding flavonol synthase (FLS, [EC: 1.14.20.6]), one of which follows the same trend as most of the DEGs on the flavonol-related pathway and continues to rise after UV-B radiation as well as exogenous ABA, yet there is a DEG that is up-regulated after UV-B radiation but declines after exogenous ABA (Table 3). The similar trends of these DEGs after UV-B treatment and exogenous ABA are consistent with the results of GO analysis, indicating that exogenous ABA is effective against the fluctuation of DEGs due to UV-B radiation. Furthermore, the vast majority of DAFs in both flavonoid-related pathways were flavonols, which is also consistent with the results of Figure 4D,E.

### 2.6. Correlation Network Analysis of Key DFs and DEGs in the Flavonoid Synthesis Pathway

**Figure 7:**
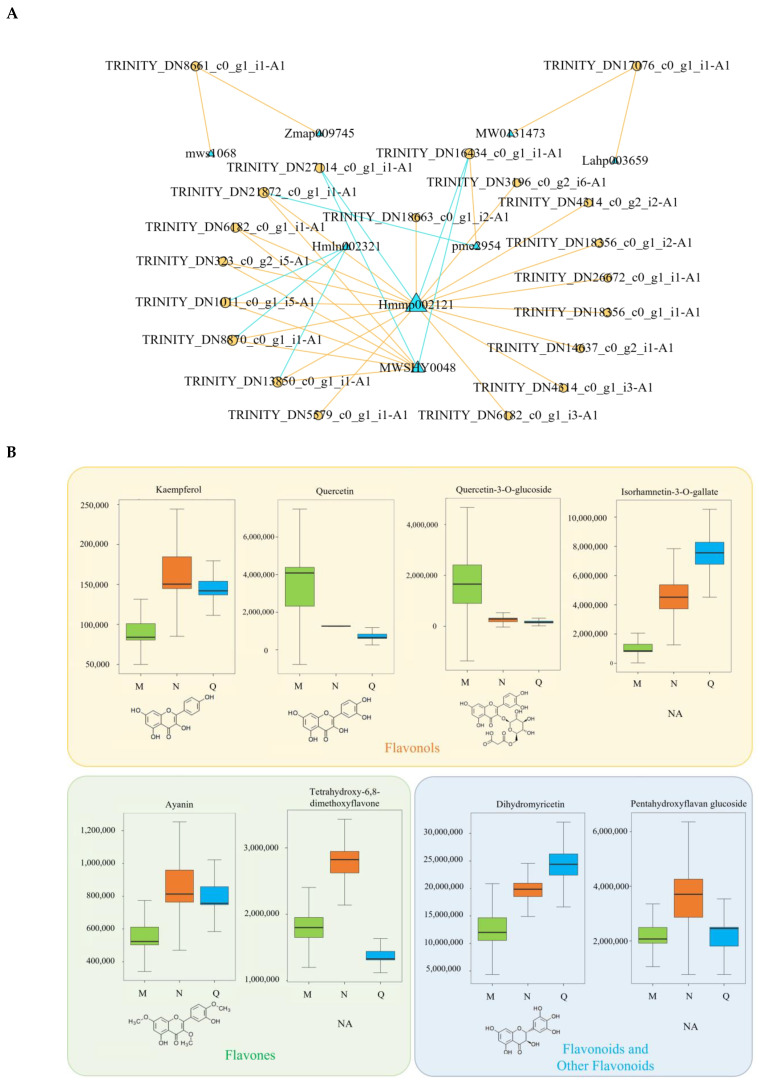

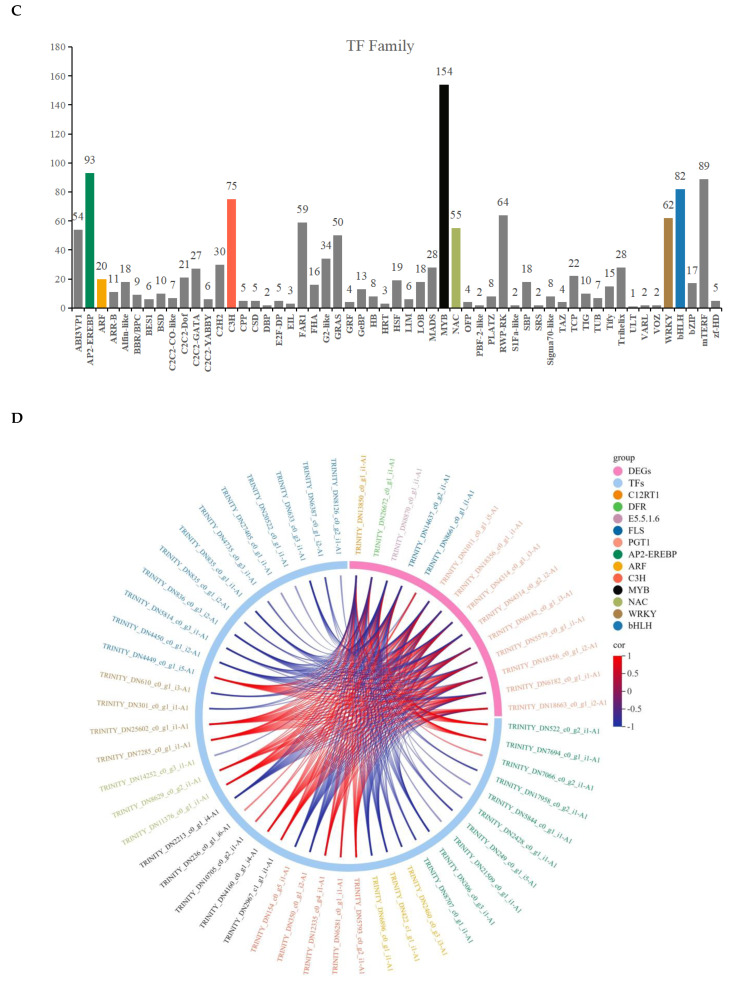
Correlation analysis of key DFs and DEGs. (**A**) Correlation network diagram between DFs and DEGs under UV-B and exogenous ABA treatments. The critical value for correlation analysis was r^2^ value ≥ 0.8 and *p* value < 0.05; blue triangles represent DFs, yellow circles represent DEGs, yellow lines indicate positive correlations, and blue lines indicate negative correlations. (**B**) Box plots of the expression of key DFs under UV-B and exogenous ABA treatments. Box plots of the expression of key DFs under UV-B and exogenous ABA treatments. (**C**) Statistics on the number of species of TFs that have been detected in *R. chrysanthum*. (**D**) Correlation analysis and chord plots of key differential TFs with enzymes involved in differential accumulation in the flavonoid pathway. Red lines indicate positive correlations and blue lines indicate negative correlations.

To investigate the DFs that play major roles after UV-B and exogenous ABA treatments and the related genes involved in them, correlation network analyses were performed on the DFs and DEGs that appeared to be co-differentially accumulated after the two experimental treatments, as well as the key DFs and DEGs in the flavonoid synthesis pathway (Figure 7A; Supplementary Table S3). The results showed a close association between 8 DFs and 20 DEGs, most of which were flavones and flavonols. Isorhamnetin-3-O-gallate (Hmmp002121) was involved in the most DEGs—a total of 18, 16 of which were positively correlated with it, and two of which were negatively correlated with it. Dihydromyricetin (MWSHY0048) is involved in 8 DEGs, of which it is positively correlated with 6 DEGs and negatively correlated with 2 DEGs. In addition, isorhamnetin-3-O-gallate and dihydromyricetin showed similar trends after UV-B and exogenous ABA treatments, and exogenous ABA further elevated the content of both of them, which increased after stimulation by UV-B (Figure 7B). It is shown that these two DFs are important UV-B-absorbing complexes for the cowpea rhododendron and contribute significantly to the defense of *R. chrysanthum* against UV-B stress.

A total of 55 families of *R. chrysanthum* TFs (transcription factors) were detected in this experiment, most of which were MYB (154), AP2-EREBP (93), mTERF (89), bHLH (82), and C3H (75) (Figure 7C). In order to make progress in revealing the regulatory mechanisms of transcription factors to UV-B stress in *R. chrysanthum*, TFs that showed differential changes after UV-B stress were correlated with differential enzymes in the flavonoid synthesis pathway (Figure 7D). Among them, MYB, NAC, and C3H were mainly positively correlated with differential enzyme genes in the flavonoid synthesis pathway, and AP2-EREBP, ARF, and bHLH were negatively correlated (Supplementary Table S4). After UV-B radiation, the expression of most of the MYB transcription factor genes increased, and most of the other key transcription factors associated with *R. chrysanthum* flavonoid biosynthesis pathway showed a decreasing trend (Supplementary Figure S5). Notably, KEGG enrichment analysis of these key TFs revealed that the TFs were mainly concentrated in plant hormone signal transduction, which laterally corroborated that plant hormones play an important role in UV-B resistance in *R. chrysanthum* (Supplementary Figure S6). An MYB (TRINITY_DN2213_c0_g1_i4-A1) that appears in phenylpropanoid biosynthesis was also found. Since phenylpropanoid biosynthesis is an important upstream pathway for flavonoid synthesis, it is highly likely that MYB (TRINITY_DN2213_c0_g1_i4-A1) is a central transcription factor in the regulation of flavonoid synthesis in response to UV-B stress in *R. chrysanthum*.

In conclusion, *R. chrysanthum* actively responds to UV-B stress by regulating the dynamics of its own flavonoids, especially flavones and flavonols. Exogenous ABA was able to modulate some key DFs, such as isorhamnetin-3-O-gallate and dihydromyricetin, to further respond to UV-B, and was effective in mitigating the damage produced by UV-B on *R. chrysanthum*.

## 3. Discussion

Recently, plants have encountered escalating stress levels owing to the slow widening of the ozone layer gap, resulting in a significant surge in ultraviolet radiation reaching the Earth’s surface [34]. Nonetheless, plants have evolved distinct resistance strategies to adjust to their growth surroundings as part of their ongoing evolutionary journey [35]. Research indicates that exposure to UV-B rays leads to decreased leaf size, curled leaves, stunted growth of the plant, and a related decline in root strength [5,36]. Plants ensure their survival in the severe conditions they encounter by adapting.

As a typical plant that survives for a long time in a high-altitude and high-radiation environment, the study of the response mechanism of *R. chrysanthum* to UV-B stress is of great significance for the study of plant response to UV-B stress. Mechanisms exist to analyze the UV-B resistance of *R. chrysanthum* from transcriptomic and phosphorylated proteomic analyses, and it was found that its ABA signaling can regulate stomatal closure through associated phosphorylated proteins in order to adapt to changes in UV-B content in its environment [26]. Research in metabolomics and proteomics revealed that exposure to UV-B rays facilitated the transformation of primary metabolites into phenolics within *R. chrysanthum* [24]. In summary, *R. chrysanthum* is able to adapt to UV-B stress through a series of metabolic changes as well as gene regulation.

Flavonoids are an important class of secondary metabolites in plants, and their metabolism is up-regulated with the enhancement of UV-B radiation over a certain range, acting as a protective barrier for UV absorption in plants [37,38]. Few studies have been conducted on the dynamics of flavonoids under UV-B stress in *R. chrysanthum* or on the effect of exogenous ABA in mitigating the damage caused by UV-B in *R. chrysanthum*. Therefore, in this experiment, we combined transcriptomics and metabolomics to further investigate the mechanism of response to UV-B stress in *R. chrysanthum* from the perspective of flavonoids, as well as to explore the link between exogenous ABA and its stress tolerance. The results of the study not only contribute to an in-depth understanding of the resistance mechanism of plants to environmental stresses but also provide an important scientific basis for addressing environmental issues such as global climate change and environmental pollution.

While photosynthesis serves as an energy source, non-biological stresses, notably UV-B rays, often harm plant photosynthesis, leading to the breakdown of plant PS II (photosystem II complex) proteins, reduced pigmentation, and harm to cyst-like structures [39]. Consequently, the assessment of chlorophyll fluorescence metrics and swift chlorophyll fluorescence induction kinetics accurately mirrors the energy transformation efficiency and photosynthesis capabilities of plants. Fo (minimal fluorescence) is the fluorescence level when all PSII reaction centers are open, reflecting the electron density of the PSII antenna pigment after excitation, and its value increases when the PSII antenna pigment is damaged [40]. The results of this study showed that the Fo value of *R. chrysanthum* increased after UV-B radiation, which indicated that UV-B radiation caused some damage to its leaf PSII reaction center and reduced the electron density of the PSII antenna pigment after excitation, leading to an increase in Fo. And, after exogenous ABA treatment, this trend of increasing Fo value due to UV-B radiation was changed, and the Fo value decreased, indicating that ABA can repair the damage caused by UV-B to the PSII antenna pigment of *R. chrysanthum* (Figure 1E,F; Table 1). It has been shown that high concentrations of Mn can significantly reduce the Fv/Fm and Y (II) values of *Celosia argentea Linn* and decrease its photosynthetic efficiency [41]. Low temperatures significantly reduced wheat Fv/Fm values and they did not return to a normal level seven days after treatment [42]. The results of the present study are in agreement with the previous findings that the Fv/Fm and Y(II) values of *R. chrysanthum* were significantly reduced (by 80% and 28%, respectively) after UV-B radiation compared to those before exposure to UV-B radiation (Figure 1A–D; Table 1). It indicates that UV-B radiation impaired the potential active center of PSII in the leaves of *R. chrysanthum*, inhibited the primary reaction of photosynthesis, and affected the photosynthetic electron transfer process. Whereas, Fv/Fm values and Y(II) values were elevated after exogenous ABA. The OJIP curve can further reflect the changes in various aspects of the photosystem of plants after stress [43,44,45,46,47]. This disruption of the photosystem of *R. chrysanthum* by UV-B radiation and the mitigating effect of exogenous ABA were equally verified in the results of the OJIP curves, which showed a degree of disruption of the donor side and the recipient side of the photosystem of *R. chrysanthum* by UV-B radiation (Figure 2). In contrast, exogenous ABA can target the damage caused by UV-B for effective repair. It is noteworthy that, although ABA mitigated the UV-B damage to PS II to some extent, the changes in Fo or Y(II) values were not significant, and the same results were observed in the OJIP curves. This suggests that, in addition to gene expression and flavonoid accumulation, other mechanisms may be involved in the plant response to UV-B. Reactive oxygen species (ROS) are direct products of UV-B radiation that can damage cellular structures. Plants scavenge excess ROS from the photosystem by enhancing their antioxidant defense system, including superoxide dismutase (SOD), catalase (CAT), and glutathione reductase (GR) [48]. It has been suggested that plants may also avoid photoinhibition by reducing the uptake of high-energy photons by photosynthetic organs through photoprotective mechanisms such as nonphotochemical quenching (NPQ) [49]. Other phytohormone plants, such as jasmonic acid (JA), may also be involved in regulating plant response to UV-B by affecting cell signaling pathways to induce corresponding adaptive changes [50]. These mechanisms have the potential to synergize with ABA and flavonoids to mitigate UV-B damage to plants, and the response mechanisms deserve further investigation.

Widely-targeted metabolomics integrates the advantages of both non-targeted and targeted metabolite detection technologies, utilizing high-resolution mass spectrometry for ultra-high-coverage secondary mass spectrometry scanning of the samples, which is characterized by high throughput, ultra-sensitivity, wide coverage, and accurate qualitative and quantitative features and has gradually become the mainstream metabolite analysis technology at present [51]. In this experiment, metabolomics combined with transcriptomics was utilized to detect a total of 487 flavonoid metabolites in *R. chrysanthum*. A total of nine flavonoids were measured in this experiment, the vast majority of which were flavones (41%), flavonols (29%), and flavanones (9%) (Figure 3D), which is similar to the results of previous experiments [52]. After UV-B radiation, most of the flavonoid contents of *R. chrysanthum* increased, with flavone, flavonol, and flavanone contents increasing in the majority of species (Figure 4D). Similar accumulations of dihydroflavonoids, flavonols, and flavanols were found in wheat after ozone stress in previous reports [53]. These classes of flavonoids have been reported to function in plant vesicles or chloroplasts for effective scavenging of hydrogen peroxide (H_2_O_2_) and monoclinic oxygen to mitigate the damage caused by stress conditions on their own [54]. Not only that, after exogenous ABA, flavones and flavonols likewise became the majority of up-regulated DFs (Figure 4D). This suggests that ABA may enhance the response of *R. chrysanthum* to UV-B stress by further regulating the changes of flavones and flavonols.

The transcriptomics results showed that the DEGs produced after UV-B radiation and exogenous ABA to *R. chrysanthum* were all equally predominant in cellular process, metabolic process, cellular anatomical entity, and catalytic activity (Supplementary Figure S4). The similar distribution of DEGs after these two treatments suggests that exogenous ABA is indeed capable of targeting and repairing the damage caused by UV-B radiation to *R. chrysanthum* at the biomolecule level. A large number of research studies related to FLS and DFR in plants exist [55,56,57,58]. The present study is consistent with previous experimental results that the expression of FLS- and DFR-associated DEGs was promoted after UV-B radiation. And studies have shown that the addition of phytohormones can effectively promote the up-regulation of FLS and DFR [59,60,61]. The same results were obtained in the present study after the application of exogenous ABA, which was able to further promote the up-regulation of FLS and DFR (Table 3). There are few reports on the response of PGT1, E5.5.1.6, or C12RT1 to abiotic stresses, and it is hypothesized that changes in these enzymes are a unique mechanism of response to UV-B in *R. chrysanthum*, deserving of further study. In the flavonoid-related pathway of the present study, the changes in DEGs were not completely consistent with those in DAFs. It is hypothesized that it may be that genes are generally more sensitive to environmental stimuli than secondary metabolism, or that these DEGs are not in one-to-one correspondence with DAFs, resulting in inconsistent trends in their changes but similar trends in the changes between the DEGs themselves and between the DAFs themselves. To some extent, the aforementioned DEGs play an important role in the flavonoid regulatory network.

While transcription factors have no impact on DFs, they have the capability to either directly or indirectly stimulate the promoters of genes linked to flavonoid biosynthesis, thus controlling the ultimate accumulation of flavonoids [62,63]. In order to make progress in revealing the regulatory mechanisms of transcription factors to UV-B stress in *R. chrysanthum*, TFs that showed differential changes after UV-B stress were correlated with differential enzymes in the flavonoid synthesis pathway (Figure 7D). Among them, MYB, NAC, and C3H were mainly positively correlated with differential enzyme genes in the flavonoid synthesis pathway, and AP2-EREBP, ARF, and bHLH were negatively correlated. Earlier research indicates that external phytohormones markedly increased MYB44 expression in oilseed rape seeds through bHLH3 transgenic apple in similar cultures, affecting anthocyanin synthesis genes and external MeJA [64,65]. Research indicates that the WRKY transcription factor is capable of elevating the expression levels of numerous isoflavonoid biosynthesis genes [66,67]. In addition to these common transcription factors, the present study found that transcription factors such as NAC, EREBP, and ARF also play important roles in the pathway related to the synthesis of *R. chrysanthum* flavonoids, which is dragged to its own specific regulatory mechanism and deserves further study.

In the present study, dihydromyricetin was found to be consistently elevated both after UV-B radiation and exogenous ABA treatment and was closely related to a variety of key flavonoid DEGs that will have been measured (Figure 7A,B). Previous findings have shown that dihydromyricetin might be linked to a variety of molecules implicated in apoptosis, oxidative stress, and inflammation [68]. Dihydromyricetin is able to increase cellular mitochondrial membrane potential while decreasing Bax levels and cysteine asparagin activation to assist cells in responding to UVA damage to themselves [69]. Research indicates that DMY enhances glucose processing, reduces oxidative stress, and prevents apoptosis through the AMPK/glucose transporter 4 pathway in MG-activated PC12 cells [70]. Current studies on the role played by dihydromyricetin in plants have centered around dihydromyricetin in Ampelopsis grossedentata, and these studies have shown that dihydromyricetin has strong antioxidant properties in plants [71,72]. However, the role of dihydromyricetin in plant response to UV-B stress has rarely been reported. Taken together, these analyses suggest that exogenous ABA may assist *R. chrysanthum* to further elevate dihydromyricetin content to regulate its own oxidative stress due to UV-B and enhance its own glucose metabolism activity, thus obtaining stronger UV-B resistance. However, the role played by isorhamnetin-3-O-gallate in planta and its mechanism of action in response to UV-B have not been reported yet. In addition, isorhamnetin-3-O-gallate and dihydromyricetin showed similar changes in this experiment, so isorhamnetin-3-O-gallate and dihydromyricetin may have similar roles in apples in response to UV-B stress, which is worthy of further study.

## 4. Materials and Methods

### 4.1. Material Preparation and Experimental Treatments

*R. chrysanthum* from the Changbai Mountains (40.10° N, 100.10° E) were used as experimental subjects and were cultivated in an intelligent artificial climate chamber simulating alpine ecology using 1/4 MS solid medium. The cultivation conditions were set as alternating cycles of light and darkness of 14 h of light and 10 h of darkness, a daytime temperature of 18 °C and a nighttime temperature of 16 °C, and the photon flux density was controlled at 50 µmol (photon) $m^{-2}$ $s^{-1}$. The final experimental treatments selected for this experiment were *R. chrysanthum* seedlings that were cultured up to 8 months with similar growth and subsequently subjected to the appropriate experimental treatments.

**Figure 8:**
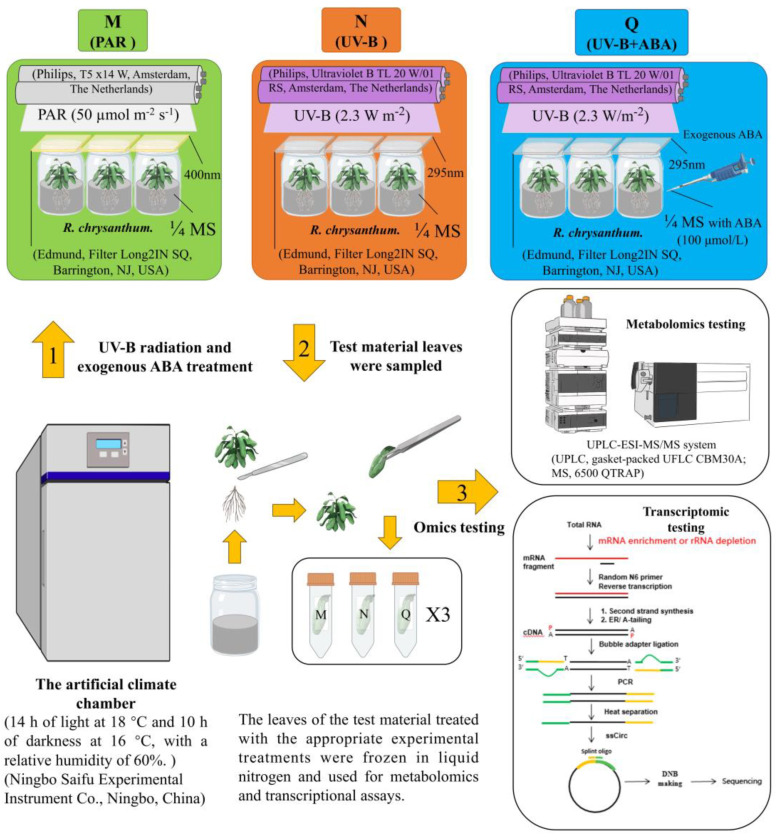
Flowchart of experimental treatment of *R. chrysanthum*.

UV-B radiation and exogenous ABA treatments were referred to previous experiments in our laboratory with minor modifications [24,25,26,73]. Seedlings of *R. chrysanthum*, identical in growth phase, were chosen for experimental purposes. Three distinct treatment groups were documented, labeled as M (PAR), N (UV-B), Q (UV-B + ABA), with each treatment cluster established through three biological duplications (*n* = 3). Cultivation of groups M and N involved using 1/4 MS solid medium for *R. chrysanthum* seedlings, while group Q utilized 1/4 MS solid medium supplemented with an additional ABA (100 µmol/L) for *R. chrysanthum* seedlings, subsequently housed in an intelligent artificial climate chamber for 6 days post-transplant. The experimental materials in group M were irradiated with PAR (50 µmol (photon) $m^{-2}$ $s^{-1}$) for 2 days, 8 h per day. The experimental materials in groups N and Q were subjected to UV-B radiation (2.3 W/m^2^) for 2 days, lasting 8 h per day. The experiment involved conducting UV-B radiation using synthetic UV-B lamps (Philips, Ultraviolet-B TL 20 W/01 RS, Amsterdam, the Netherlands), emitting wavelengths between 280 and 320 nm. The irradiance of the samples treated with UV-B was determined in accordance with the transmission function of the long-pass filter, utilizing a UV intensity meter (Sentry Optron-ICS Corp., ST-513, SHH, New Taipei City, China) and a light meter (TES Electrical Electronic Corp., Tes-1339 Light Meter Pro., Taipei, China). To eliminate interference, a 295 nm filter (Edmund, Filter Long 2IN SQ, Barrington, NJ, USA) was positioned over the *R. chrysanthum*s exposed to UV-B radiation, while a 400 nm filter (Edmund, Filter Long 2IN SQ, Barrington, NJ, USA) was used on the *R. chrysanthum*s unexposed to UV-B radiation for radiation filtration. Finally, the leaves of the materials treated with the corresponding experimental treatments were frozen in liquid nitrogen for subsequent metabolomics and transcriptomics assays (Figure 8).

### 4.2. Widely-Targeted Metabolome Testing

Metware Biotech Inc. (Wuhan, China) was commissioned to analyze the samples by UPLC-MS/MS. Widely-targeted metabolomics assays as well as the instrumentation and reagent types involved in the experiments were strictly based on previous experimental descriptions [24]. In summary, MWDB (metware database) qualitatively examined the mass spectrometry’s primary and secondary spectra, Analyst 1.6.3 software both qualitatively and quantitatively assessed the gathered data, and Multial Quant 3.0.2 software integrated and adjusted the mass spectrometry findings for chromatographic peaks and ultimately acquired the metabolite-related information. DMS (differential metabolites) were screened according to the criteria of VIP > 1 and FC ≥ 1.5/FC ≤ 0.67 and annotated into the KEGG database, and, finally, the flavonoid metabolic pathway analysis was performed by the KEGG database.

### 4.3. Transcriptomics Testing

Transcriptomics testing was performed by Huada Gene Science and Technology Research Co (Shenzhen, China). Transcriptomics analysis methods and experimental conditions were likewise performed in strict accordance with previous experimental descriptions [74]. A brief description is as follows:

RNA extraction and library construction: The samples were entrusted to Huada Gene Science and Technology Research Co. to extract the total RNA by CTAB method, and the RNA was processed to obtain the single-stranded circular DNA library.

RNA-seq and data assembly: RNA-seq was performed using the MGISEQ-2000 platform. The sequencing data were statistically analyzed with the filtering software SOAPnuke (v1.4.0), trimmomatic filtering, Bowtie2 to compare clean reads to the reference gene sequence and RSEM to calculate the expression levels of genes and transcripts.

Unigene functional annotation and classification: Utilizing the resources of seven public databases, including NT, GO, KEGG, KOG, Pfam, Swissprot, and NR, Unigene sequences were compared, information annotated, and classified using BLAST. The statistic used was q-value (adjusted *p* value) < 0.05. The study analyzed for DEGs (differentially expressed genes) by utilizing FC > 1 and q-value < 0.05.

### 4.4. Determination of Chlorophyll Fluorescence Parameters

The experimental methods refer to previous descriptions [75]. The IMAGING-PAM chlorophyll fluorescence imaging system (Heinz Walz, Germany) was selected for the determination of chlorophyll fluorescence parameters, and the leaves were dark-adapted for 30 min before testing. Fo (minimal fluorescence) was induced by measured light, and Fm (maximum fluorescence) was produced by excitation with saturated pulsed light. When the fluorescence was reduced to Fo, fluorescence kinetics were induced using photochemical light (1600 μmol·m^−2^·s^−1^), and saturating pulses were turned on at 20 s intervals to determine the maximum fluorescence yield, Fm, when all the PS II reaction centers were off, and the actual fluorescence intensity, F, at any given time. The final parameters measured include Fv/Fm (maximum efficiency of PSII photochemistry), Y(II) (actual efficiency of PSII photochemistry), and Fo (initial fluorescence) (Table 4).

### 4.5. Determination of OJIP Curve

The OJIP curves of *R. chrysanthum* leaves were determined using Handy-PEA (Hansatech, UK) after adequate dark adaptation for 30 min. The OJIP curves were induced by 3000 μmol·m^−2^·s^−1^ of red light with a measurement time of 1 s. The OJIP curves were standardized for the O-J section, O-P section, and O-points by referring to the previous method [76]. Point O is the minimum fluorescence value, point K is the 300 µs fluorescence value, point J is the 2 ms fluorescence value, and point P is the maximum fluorescence value.

Calculation of fluorescence parameters:

*V*_t_ = (*F*_t_ − *F*_o_)/(*F*_m_ − *F*_o_); *V*_J_ = (*F*_J_ − *F*_o_)/(*F*_m_ − *F*_o_); △*W*_t_ = (*W*_t_) _treatment_ − (*W*_t_) _CK_; △*V*_t_ = (*V*_t_) _treatment_ − (*V*_t_) _CK._

### 4.6. Statistical Analysis

OPLS-DA (orthogonal partial least squares discriminant analysis) was centered after log2 transformation of the raw data, and then the MetaboAnalystR package in R software (1.0.1) (www.r-project.org (accessed on 9 July 2022)) OPLSR. Anal function was utilized for the analysis. The HCA (hierarchical cluster analysis) results of samples and metabolites were presented as heatmaps. The HCA were carried out by R package ComplexHeatmap (2.9.4). Identified metabolites were annotated using KEGG compound database (http://www.kegg.jp/kegg/compound/ (accessed on 9 April 2022)), annotated metabolites were then mapped to KEGG pathway database (http://www.kegg.jp/kegg/pathway.html (accessed on 12 July 2022)). The function package pandas (0.23.4) and the analytic package Python (3.6.6) were used to create boxplots. For the statistical analysis, IBM SPSS statistical software (IBM SPSS Statistics 26) (https://www.ibm.com/analytics/spss-statistics (accessed on 28 July 2022)) was employed. Significantly different results are indicated by different letters. One-way analysis of variance (ANOVA) and Pearson’s correlation tests at the 5% level were applied to analyze the data.

## Figures and Tables

**Table 1:** Effect of exogenous ABA on chlorophyll fluorescence characteristics of leaves of *R. chrysanthum* under UV-B stress.

Treamments	Fv/Fm	Y(II)	Fo
PAR	0.80 ± 0.002 a	0.18 ± 0.008 a	0.1065 ± 0.0016 a
UV-B	0.57 ± 0.017 c	0.13 ± 0.012 b	0.1072 ± 0.0036 a
UV-B + ABA	0.68 ± 0.029 b	0.17 ± 0.031 ab	0.1056 ± 0.0050 a

Note: Different letters denote significant differences between treatments (*p* < 0.05).

**Table 2:** *R. chrysanthum* leaves sample sequencing data evaluation statistics.

Sample	Total Raw Reads (M)	Total Clean Reads (M)	Q20 (%)	Q30 (%)	GC Content (%)
M1	43.69	42.55	98.11	93.41	43.64
M2	43.69	42.34	97.83	92.4	43.73
M3	43.69	42.39	98.15	93.57	43.63
N1	43.69	42.39	98.14	93.52	43.84
N2	43.69	42.37	97.96	92.87	43.86
N3	45.44	43.46	98.43	94.51	43.72
Q1	45.44	43.3	98.28	94.09	44.01
Q2	43.69	42.35	98.05	93.16	43.79
Q3	43.69	42.38	97.89	92.61	43.85

**Table 3:** Information about genes involved in enzymes altered in flavonoid-related pathways.

Gene Annotation	Gene ID	M FPKM	N FPKM	Q FPKM	log2(N/M)	Type (MN)	log2(Q/N)	Type (NQ)
E5.5.1.6	TRIITY_DN8870_c0_g1_i1-A1	40.09	57.31	60.77	0.62	UP	0.08	UP
C12RT1	TRIITY_DN13850_c0_g1_i1-A1	6.47	13.35	18.85	1.18	UP	0.50	UP
PGT1	TRIITY_DN1011_c0_g1_i5-A1	34.83	60.62	86.63	0.95	UP	0.51	UP
	TRIITY_DN18356_c0_g1_i1-A1	9.92	39.38	71.62	2.13	UP	0.86	UP
	TRIITY_DN18356_c0_g1_i2-A1	8.70	38.21	81.37	2.28	UP	1.09	UP
	TRIITY_DN18663_c0_g1_i2-A1	5.56	18.87	44.13	1.94	UP	1.23	UP
	TRIITY_DN4314_c0_g1_i3-A1	26.71	51.52	81.98	1.08	UP	0.67	UP
	TRIITY_DN4314_c0_g2_i2-A1	6.80	20.16	31.40	1.74	UP	0.64	UP
	TRIITY_DN5579_c0_g1_i1-A1	23.16	64.69	132.54	1.62	UP	1.03	UP
	TRIITY_DN6182_c0_g1_i1-A1	5.94	31.04	59.57	2.57	UP	0.94	UP
	TRIITY_DN6182_c0_g1_i3-A1	5.36	18.55	42.34	1.98	UP	1.19	UP
FLS	TRIITY_DN14637_c0_g2_i1-A1	9.45	18.83	23.55	1.11	UP	0.32	UP
	TRIITY_DN8661_c0_g1_i1-A1	10.63	43.70	38.56	2.12	UP	−0.18	DOWN
DFR	TRIITY_DN26672_c0_g1_i1-A1	11.70	37.19	66.67	1.82	UP	0.84	UP

**Table 4:** Chlorophyll fluorescence parameters and calculation equations.

Chlorophyll Fluorescence Parameters	Formula
Maximum efficiency of PSII photochemistry (Fv/Fm)	(Fm − Fo)/Fm
Actual efficiency of PSII photochemistry (Y(II))	(Fm′ − F)/Fm′

## Data Availability

The data used in this study are available from the corresponding author on submission of a reasonable request.

## Supplementary Materials

The supporting information can be downloaded at: https://www.mdpi.com/article/10.3390/ijms25105248/s1.

## Abbreviations

*R. chrysanthum*	*Rhododendron chrysanthum* Pall.
ABA	Abscisic acid
ANOVA	Analysis of variances
UPLC-MS/MS	Ultra-performance liquid chromatography tandem mass spectrometry
MWDB	Metware database
Q-value	Adjusted *p* value
UV-B	Ultraviolet radiation b
PAR	Photosynthetic active radiation
DMS	Differential metabolites
DFs	Different flavonoids
DEGs	Differentially expressed genes
PSII	Photosystem II
Fo	Minimal fluorescence
PQ	Plastoquinone
QA	The primary quinone acceptor
QB	The secondary quinone acceptor
OEC	Oxygen evolving complex
GO	Gene Ontology
KEGG	Kyoto Encyclopedia of Genes and Genomes
H_2_O_2_	Hydrogen peroxide
OPLS-DA	Orthogonal partial least squares discriminant analysis
FC	Fold change
VIP	Variable important in projection
FPKM	Transcript fragments per kilobase per million mapped reads
E5.5.1.6	Chalcone isomerase
C12RT1	Flavanone 7-O-glucoside 2″-O-beta-L-rhamnosyltransferase
DFR	Dihydroflavonol 4-reductase
PGT1	Phlorizin synthase
TFs	Transcription factors
