# Supplementary material for: Molecular Mechanism of Exogenous ABA to Enhance UV-B Resistance in Rhododendron chrysanthum Pall. by Modulating Flavonoid Accumulation
Source: Int J Mol Sci. 2024 May 11;25(10):5248. doi: 10.3390/ijms25105248 (PMC11121613; doi:10.3390/ijms25105248)
Supplement: Supplementary file 1 [file ijms-25-05248-s001.zip › Supplementary Figure.pdf]

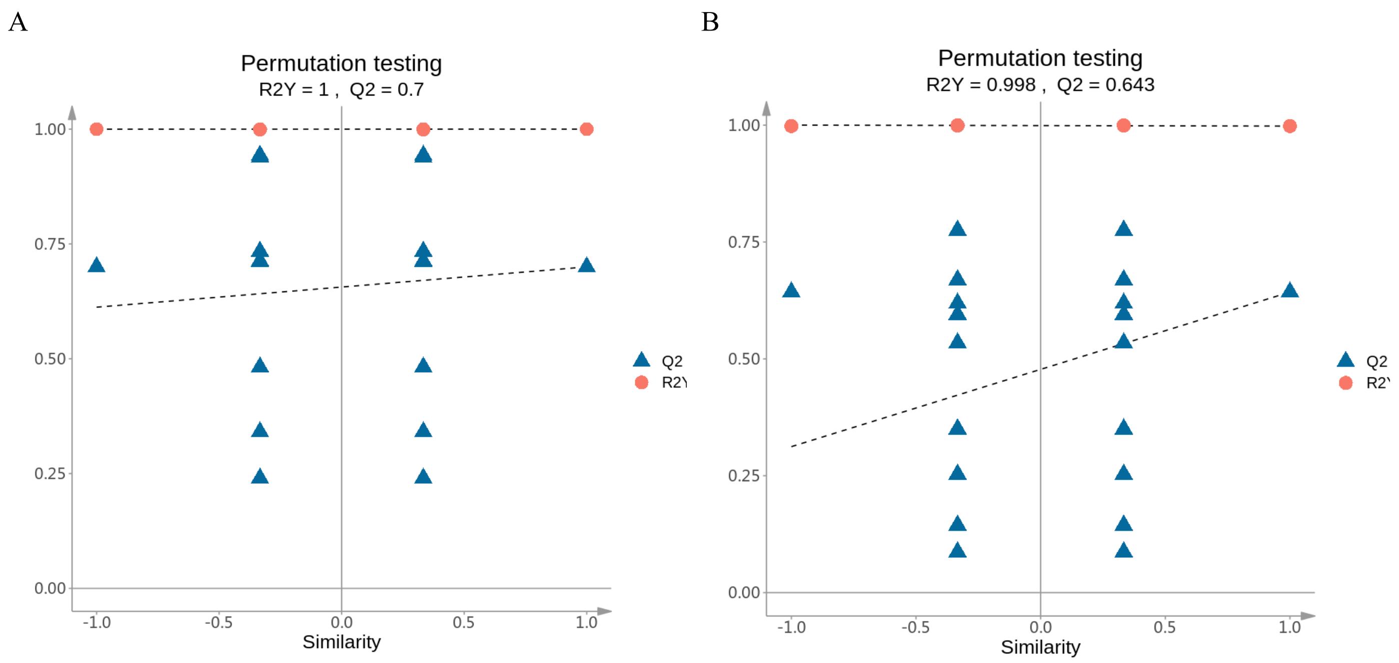

Supplementary Figure S1: A,OPLS-DA evaluation model (MvsN) ; A,OPLS-DA evaluation model (NvsQ) R^2X and R^2Y denote the explanatory rate of the constructed model on the X and Y matrices, respectively, and Q^2 denotes the predictive power of the model. A model is generally considered valid when $Q^2 > 0.5$.

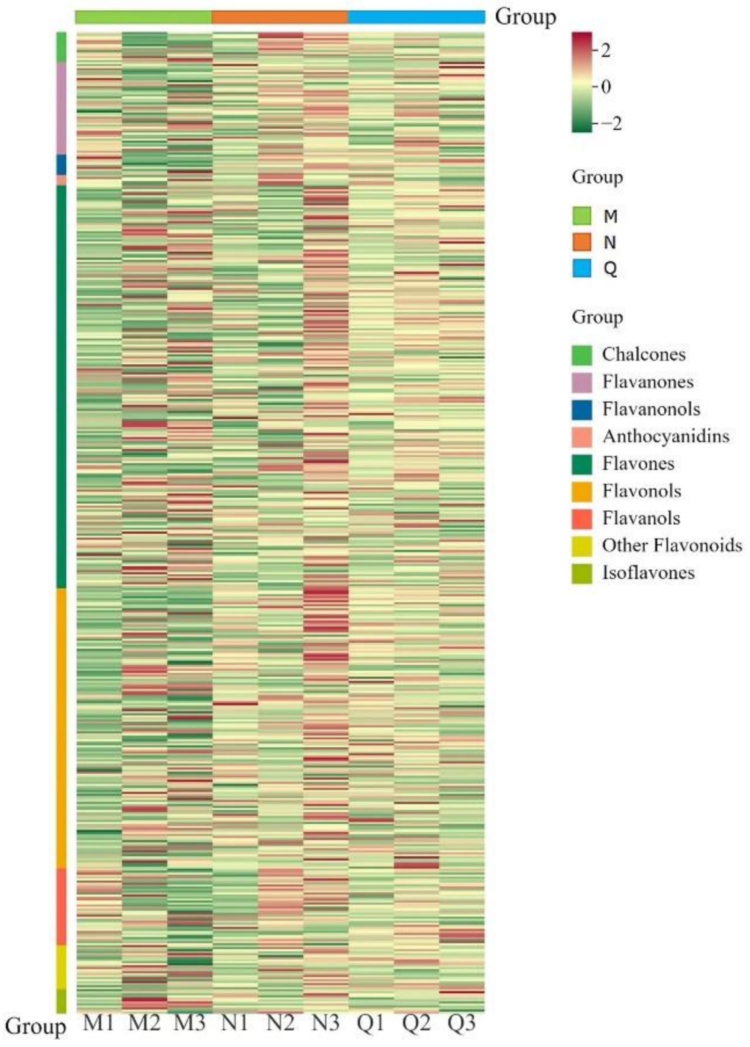

Supplementary Figure S2: Clustering heatmap of the detected 487 flavonoids.

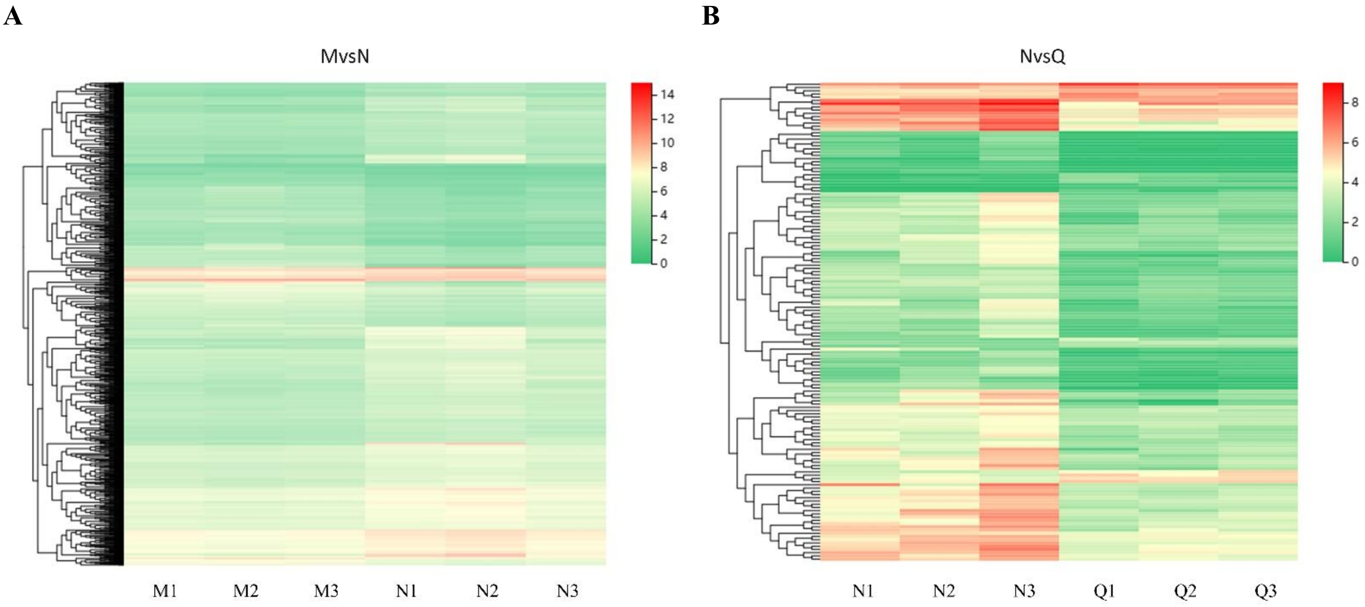

Supplementary Figure S3: A, Hierarchical clustering analysis of DEGs in Groups M and N; B, Hierarchical cluster analysis of DEGs in Groups N and Q.

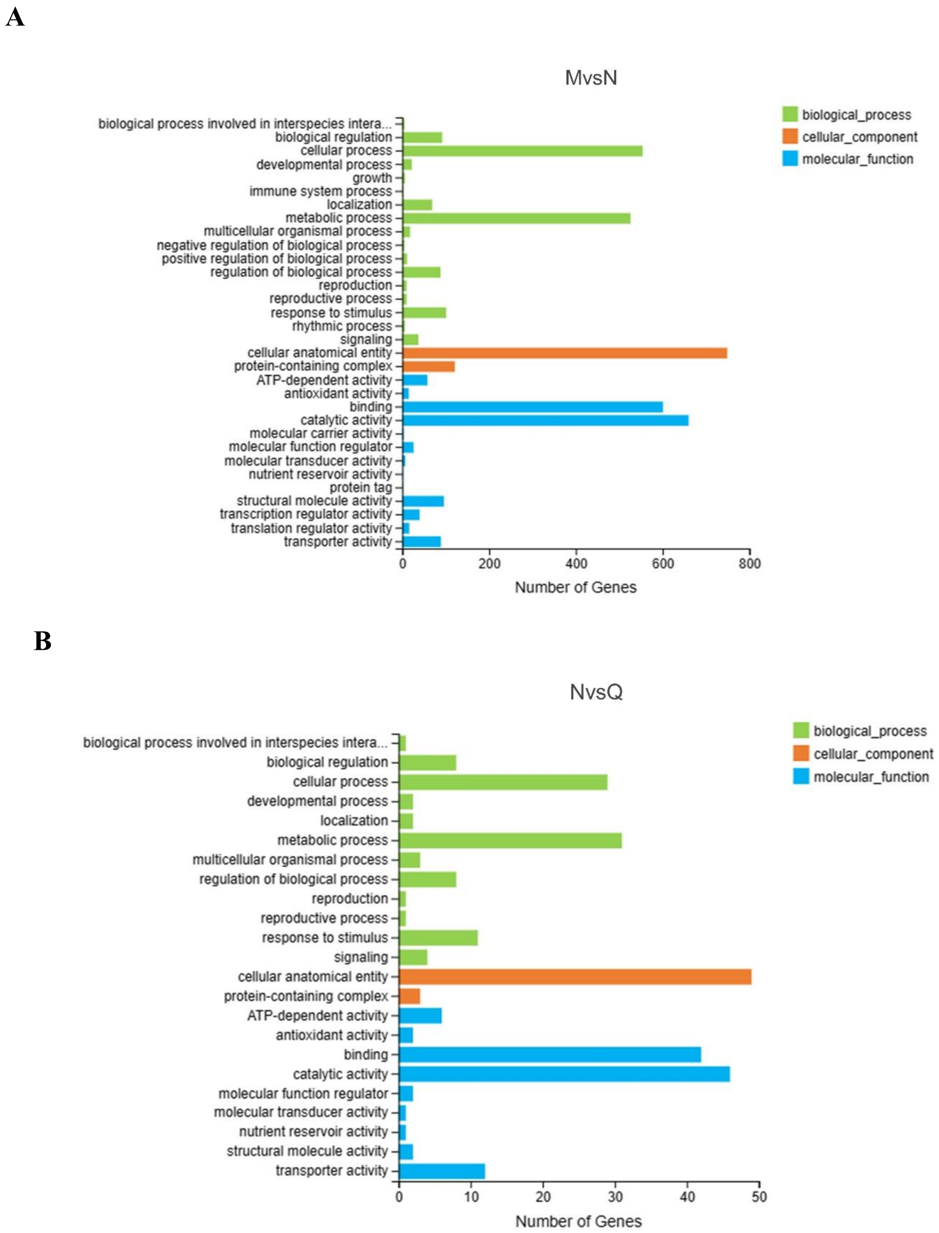

Supplementary Figure S4: A, Functional classification of GO term enrichment analysis of DEGs in Group M and Group

N comparison.; B, Functional classification of GO term enrichment analysis of DEGs in Group N and Group Q comparison.

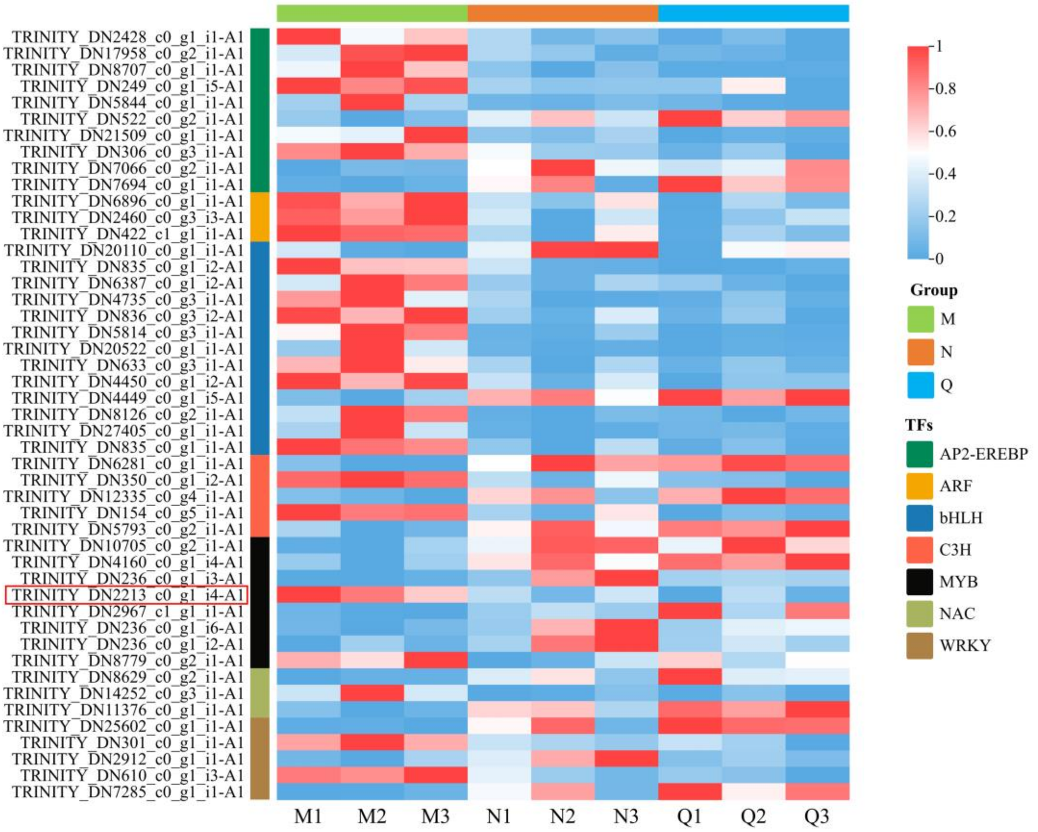

Supplementary Figure S5: Clustering heatmap of key TFs regulating flavonoid-related pathway DEGs.

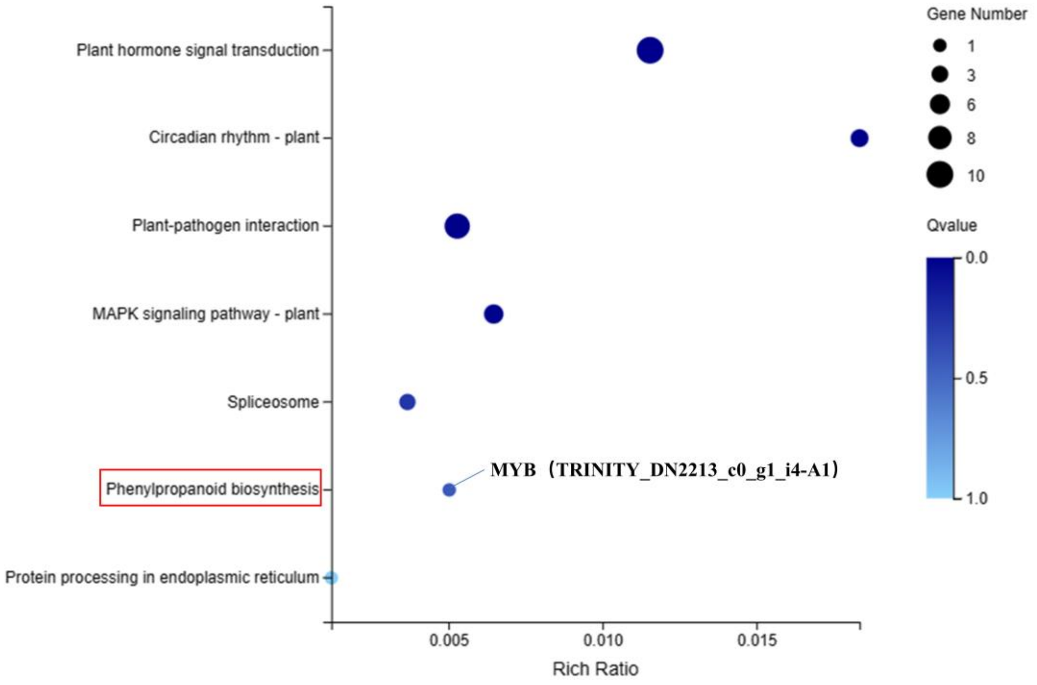

Supplementary Figure S6: Bubble map of KEGG Pathway enrichment of TFs.
